# Binding interactions of epididymal protease inhibitor and semenogelin-1: a homology modeling, docking and molecular dynamics simulation study

**DOI:** 10.7717/peerj.7329

**Published:** 2019-08-05

**Authors:** Changyu Shan, Hongwei Li, Yuping Zhang, Yuyan Li, Yingchun Chen, Wei He

**Affiliations:** 1 Department of Pharmaceutical Chemistry, The Third Military Medical University, Chongqing, China; 2 College of Pharmacy and Bioengineering, Chongqing University of Technology, Chongqing, China; 3 Department of Obstetrics and Gynecology, Southwest Hospital, The Third Military Medical University, Chongqing, China

**Keywords:** Homology modeling, Epididymal protease inhibitor, Semenogelin-1, Molecular docking, Molecular dynamics simulation

## Abstract

Epididymal protease inhibitor (EPPIN) that is located on the sperm surface and specific to the male reproductive system is a non-hormonal contraceptive target, since the binding of EPPIN with the seminal plasma protein semenogelin-1 (SEMG1) causes a loss of sperm function. Here, we investigated the binding interactions between EPPIN and SEMG1 by homology modeling, docking and molecular dynamics simulation. Since no crystal structure was reported for EPPIN, its 3D structure was constructed by homology modeling and refined by dynamics simulation, illustrating the C-terminus domain of EPPIN could bind with its N-terminus domain through the residues 30–32 and 113–116. The binding interaction of SEMG1^10-8^ peptide and EPPIN was investigated by Z-DOCK and dynamics simulation. After evaluating the models according to the calculated binding free energies, we demonstrated that C-terminus domain of EPPIN was important for the binding of SEMG1 via residues Tyr107, Gly112, Asn116, Gln118 and Asn122, while residue Arg32 in N-terminus domain also had contribution for their binding interaction. Additionally, the binding pocket of EPPIN was defined according to these key residues and verified by molecular docking with reported inhibitor **EP055**, suggesting that the pocket formed by Arg32, Asn114, Asn116, Phe117 and Asn122 could be important for the design of new ligands. This study might be helpful for the understanding of biological function of EPPIN and would encourage the discovery of non-hormonal contraceptive leads/drugs in the future.

## Introduction

The use of condoms and the vasectomy operation were the classical methods for male contraception for many years (Youssef, 1993). The high use failure rate (18%) of the condom and the irreversibility of vasectomy (Guttmacher Institute, 2018) have encouraged the pursuit of new targets for the development of male hormonal (Chao & Page, 2016) and non-hormonal (Mannowetz, Miller & Lishko, 2017; O’Rand, Silva & Hamil, 2016) contraceptives, including the inhibition of sperm function (O’Rand, Silva & Hamil, 2016) and physical blockage of the production and delivery of sperm (Colagross-Schouten et al., 2017; Waller et al., 2016).

The epididymal protease inhibitor (EPPIN), an example of a rat whey acidic protein four-disulfide core gene, is located on the surface of spermatozoa. In addition to its anti-bacterial activity (Yenugu et al., 2004) and its ability to modulate the proteolytic activity of a serine protease named prostate specific antigen (O’Rand et al., 2006), EPPIN inhibits sperm motility by binding to the seminal plasma protein, semenogelin-1 (SEMG1) (Wang et al., 2005). The binding of EPPIN and SEMG1 causes a loss of sperm function manifested as a rapid decrease in internal pH and calcium levels (O’Rand & Widgren, 2012). Because EPPIN is specific to the male reproductive system and its essential function on ejaculated spermatozoa could be reversibly blocked with easy access to the target on the sperm surface, this molecule was studied as a reasonable non-hormonal contraceptive target.

The EPPIN protein is a cysteine-rich protein, which contains both Kunitz (Asp26-Lys73) and WAP domains (Cys77-Cys127) (Richardson et al., 2001). Mutagenesis studies have demonstrated that Cys102, Tyr107, and Phe117 located at the Kunitz domain were of great significance for the binding affinity of EPPIN and SEMG1 (Silva et al., 2012). On the other hand, the truncation experiments with a series of SEMG1 peptides showed that the domain from 229 to 247 was considered as the EPPIN binding domain and Cys239 was the key residue (Silva, Hamil & O’Rand, 2013).

The crystalline structures of EPPIN and SEMG1 have not been reported. A computer model of EPPIN-SEMG1 was used to screen for hits, and a series of small organic compounds based on this strategy were found (Hirano et al., 2001; Silva, Hamil & O’Rand, 2013). In particular, **EP055** was reported as a potential male contraceptive since it should provide a reversible, short-lived pharmacological alternative (O’Rand et al., 2018) in the cynomolgus males. However, the detailed interactions of EPPIN and SEMG1 as well as the structural dynamic changes of EPPIN-SEMG1 complex, which should be helpful for understanding the mechanism of male contraception and developing new male contraceptives, have not been derived. Here, we reported a study on the binding interactions between EPPIN and SEMG1 by using homology modeling, molecular docking, and molecular dynamics (MD) simulation. The results suggested that the C- and N-terminus domains of EPPIN were important for the binding of SEMG1. Additionally, we found the pocket formed by Arg32, Asn114, Asn116, Phe117 and Asn122 could be important for the design of new ligands of EPPIN.

## Materials and Methods

### Modeling of EPPIN and SEMG1

The full sequence of human EPPIN protein (EPPI_HUMAN or O95925, 133 residues) was retrieved from the UniProtKB (http://www.uniprot.org/uniprot/). After truncating the signal peptide (Met1-Gly21), the N-terminus (Pro22-Asp71) and C-terminus (Lys73-Pro133) of EPPIN protein were aligned and constructed separately by using Discovery Studio 3.0 (Chen & Weng, 2003). After BLAST searching from the NCBI server, the known crystalline structures of neutrophil elastase (PDB ID: 2Z7F, chain A) (Koizumi et al., 2008) and carboxypeptidase inhibitor SmCI (PDB ID: 4BD9, chain B) (Del Alonso et al., 2013) showed the most similar sequences alignment to the EPPIN C-terminus (38.9%) and N-terminus (60.3%), respectively. Hence, we selected these two structures to build the C- and N-terminuses of EPPIN, respectively. According to the suggestion of Disulfide Bridge from UniProtKB, 7 disulfide bridge/bonds between Cys33-Cys61, Cys40-Cys65, Cys48-Cys60, Cys54-Cys69, Cys77-Cys127, Cys86-Cys110 and Cys102-Cys123 in EPPIN were patched (Silva et al., 2012). After the structures of N- and C-terminuses were homology modeled, they were linked and further refined by Discovery Studio 3.0 (Chen & Weng, 2003).

As for SEMG1, we selected a short-truncated fragment sequences flanking Cys239 residue, namely SEMG1^10-8^, representing SEMG1^E229-Q247^. As the BLAST searching result demonstrated low identities aligning with known crystal structures, the structure of this sequence was predicted and modeled by the I-TASSER server (Yang et al., 2015) and the one with the highest *C*-score was selected for further study, since the *C*-score is a confidence score for estimating the quality of predicted models by I-TASSER, where a *C*-score of higher value signifies a model with a high confidence and vice-versa (Yang et al., 2015).

### Energy minimization and structure validation

After generating 3D models of EPPIN and SEMG1, we performed the energy minimizations using SYBYL-X 2.0 with the Powell method under AMBER7 FF99 force field and AMBER charges. The energy minimizations were terminated when the iterations reached 10,000 steps or the energy gradient less than 0.5 kcal/mol.

Structure evaluation and stereo-chemical analysis for the EPPIN models were performed by using proSA-web *Z*-scores (Wiederstein & Sippl, 2007) and PROCHECK Ramachandran plots (Laskowski et al., 1993). The visualization of the generated models was performed using the PyMOL program (Schrodinger, LLC, 2015).

### Molecular dynamics simulation

We selected the EPPIN and SEMG1^10-8^ to perform MD simulation studies. The residues were ionized within the physiological pH range (∼7.40). To determine the protonation states for histidines and other residues, Discovery Studio 3.0 (Chen & Weng, 2003) was used to predict the pK values of the residues. As calculated pK values were lower than 7.40, all histidines were predicted not to be protonated. Sidechains of Asp, Glu, Arg and Lys were charged thus Asp^−^, Glu^−^, Arg^+^ and Lys^+^, respectively, according to all the simulations.

The solvent molecules and additional ions for simulations were added using the *tleap* (Case et al., 2005) module of AMBER14 under the ff14SB force field (Maier et al., 2015). The systems were first neutralized with Na^+^ or Cl^−^, then solvated in the TIP3P water model (Jorgensen et al., 1983) and subsequently placed in a regular hexahedron box with a minimal distance of 12 Å for the solute from the box borders.

The systems were first minimized in the AMBER14 *pmemd.MPI* module in three stages. At the first two stages, the whole proteins or their backbone atoms were fixed by applying a harmonic force constant of 2 kcal/mol Å^2^ respectively, thus making the water molecules and protein side chains free to move successively. In the following stage, the restraint strength was abolished, so that the entire systems would be able to move freely.

After the minimization, the systems were gradually heated from 0° K to 300° K for a total 50 pico-seconds (ps). This step was performed using the Langevin thermostat (Izaguirre et al., 2001) with a collision frequency of 2.0 ps^−1^. Then, the pressure of systems was kept constant using a 50 ps simulation. Following this, in order to obtain a system equilibrium, a simulation of 0.5 nano-second (ns) was performed at 300° K, with constant pressure and without restriction.

Starting from the last frame of the equilibration, MD simulations for the different systems were performed, respectively. The MD simulations were ran by using the *pmemd.CUDA.MPI* module of AMBER 14. The electrostatics interaction was calculated using the particle mesh Ewald (Darden, York & Pedersen, 1993) method with an 8 Å non-bonded cutoff. The temperature and pressure of the system were kept constant during the whole MD simulations. The time interval for the MD was set as 2 femto-seconds (fs). The data were saved every 10 ps for analysis. Subsequently, 50 or more ns MD simulations were performed under 300° K and 1 atm.

The analyses of root mean square deviation (RMSD), root mean square fluctuation (RMSF), radius of gyration (*R*_g_) and atom distances were carried out with the AMBER14 module *CPPTRAJ*, VMD and PyMOL programs (Schrodinger, LLC, 2015), respectively.

### Conformations sampling for EPPIN and SEMG1

To select the most reasonable conformation of EPPIN and SEMG1 models for further studies, after the MD simulation reached equilibrium, some conformations were sampled by extracting one conformation per five ns. After initial energy minimizations using the methods introduced in the previous section (“Energy minimization and structure validation”), we selected the conformation with the lowest energy as the most reasonable model for further docking and MD simulations.

### Molecular docking study of EPPIN-SEMG1 complexes

The docking study of the EPPIN-SEMG1^10-8^ (protein-peptide) interaction was performed according to the ZDOCK module of Discovery Studio 3.0 (Chen & Weng, 2003). The selected 3D model of EPPIN and SEMG1^10-8^ were used as inputs for the receptor and the ligand, respectively. According to the previous findings (Silva et al., 2012), we selected Tyr107 and Phe117 of EPPIN and Cys239 of SEMG1^10-8^ as the binding site residues. After the predicted binding models (EPPIN-SEMG1^10-8^) were constructed by the ZDOCK module, we randomly chose three different poses from the top 50 ranks in the area with the densest docking cluster for further studies.

### MM/PBSA calculations

To calculate the binding free energies between EPPIN and SEMG1^10-8^ for three different binding conformations, 85 ns MD simulations were performed using the previous MD protocol, until the systems reached equilibrium. The binding free energies were calculated using the Molecular Mechanics Poisson–Boltzmann Surface Area (MM/PBSA) method (Chen et al., 2016, Sun et al., 2014a, 2014b) implemented in AMBER 14. For each system, 100 snapshots of the equilibrium stage were used from the MD trajectory. For each snapshot, free energy was calculated for EPPIN, SEMG1^10-8^ and the EPPIN-SEMG1^10-8^ complex using a single trajectory approach. The total binding free energy can be calculated according to the following equation (Fang et al., 2014):

(1) $$\Delta {G_{{\rm{bind}}}} = \Delta {G_{{\rm{complex}}}}-\Delta {G_{{\rm{receptor}}}}-\Delta {G_{{\rm{ligand}}}}$$

(2) $$\Delta {G_{{\rm{bind}}}} = \Delta {E_{{\rm{MM}}}} + \Delta {G_{{\rm{solv}}}}-T\Delta S$$

(3) $$\Delta {E_{{\rm{MM}}}} = \Delta {E_{{\rm{vdw}}}} + \Delta {E_{{\rm{ele}}}}$$

(4) $$\Delta {G_{{\rm{solv}}}} = \Delta {G_{{\rm{PB}}}} + \Delta {G_{{\rm{SA}}}}$$

Where Δ*E*_MM_ denotes the gas-phase interaction energy between the receptor and the ligand (including van der Waals energy contribution (Δ*E*_vdw_) and electrostatic energy contribution (Δ*E_ele_*)); Δ*G*_PB_ and Δ*G*_SA_ are the polar and nonpolar components of the de-solvation free energy, respectively; *T*Δ*S* represents the conformational entropy contribution at temperature *T*. Here, Δ*G*_PB_ was determined by the Poisson–Boltzmann approximation model, while Δ*G*_SA_ was estimated based on the solvent accessible surface area model by the method: Δ*G*_SA_ = γ × SASA + β, where the values of the constants γ and β were 0.00542 kcal·Å^−2^ and 0.92 kcal·mol^−1^, respectively, (Weiser, Shenkin & Still, 1999). The solvent probe radius and ionic strength were set to be 1.4 Å and 0.1 mM, respectively. The interior and exterior dielectric constant of MM/PBSA calculation systems was 1.0 and 80.0.

### Investigation of potential binding pockets by molecular docking studies

Surflex-Dock GeomX module in SYBYL-X 2.0 was used to investigate the potential binding pockets of EPPIN. During the progress of investigating these pockets, we selected the Multi-channel Surface mode and the important residues Gly109, Cys110, Gln111, Gly112, Asn113, Asn114 and Asn116 to generate and select binding pockets of EPPIN, respectively. The EPPIN ligand **EP055** (O’Rand et al., 2018) was docked into the binding pocket using the Surflex-Dock GeomX module. The visualization of the EPPIN-EP055 complex interaction was performed using PyMOL (Schrodinger, LLC, 2015) programs.

## Results and Discussion

### Homological model, equilibration and stability of EPPIN

As the crystalline structure is not available for EPPIN, the 3D model of EPPIN was constructed by homological modeling. The neutrophil elastase (PDB ID: 2Z7F, chain A) (Koizumi et al., 2008) was chosen as the reference structure of N-terminus because it had greater than 40% sequence identity. Similarly, the carboxypeptidase inhibitor SmCI (PDB ID: 4BD9, chain B) was selected as the template for C-terminus domain (77–133) (Del Alonso et al., 2013). The detailed sequence alignment of EPPIN C-terminus domain and its structure templates was shown in Fig. S1. As there are seven disulfide bonds in EPPIN, the alignment of the critical Cys amino acids for disulfide bonds in the candidate templates was also considered as another important factor for modeling criteria. We also used I-TASSER to build the model of each domain of EPPIN which was similar to the homological model.

**Figure 1:**
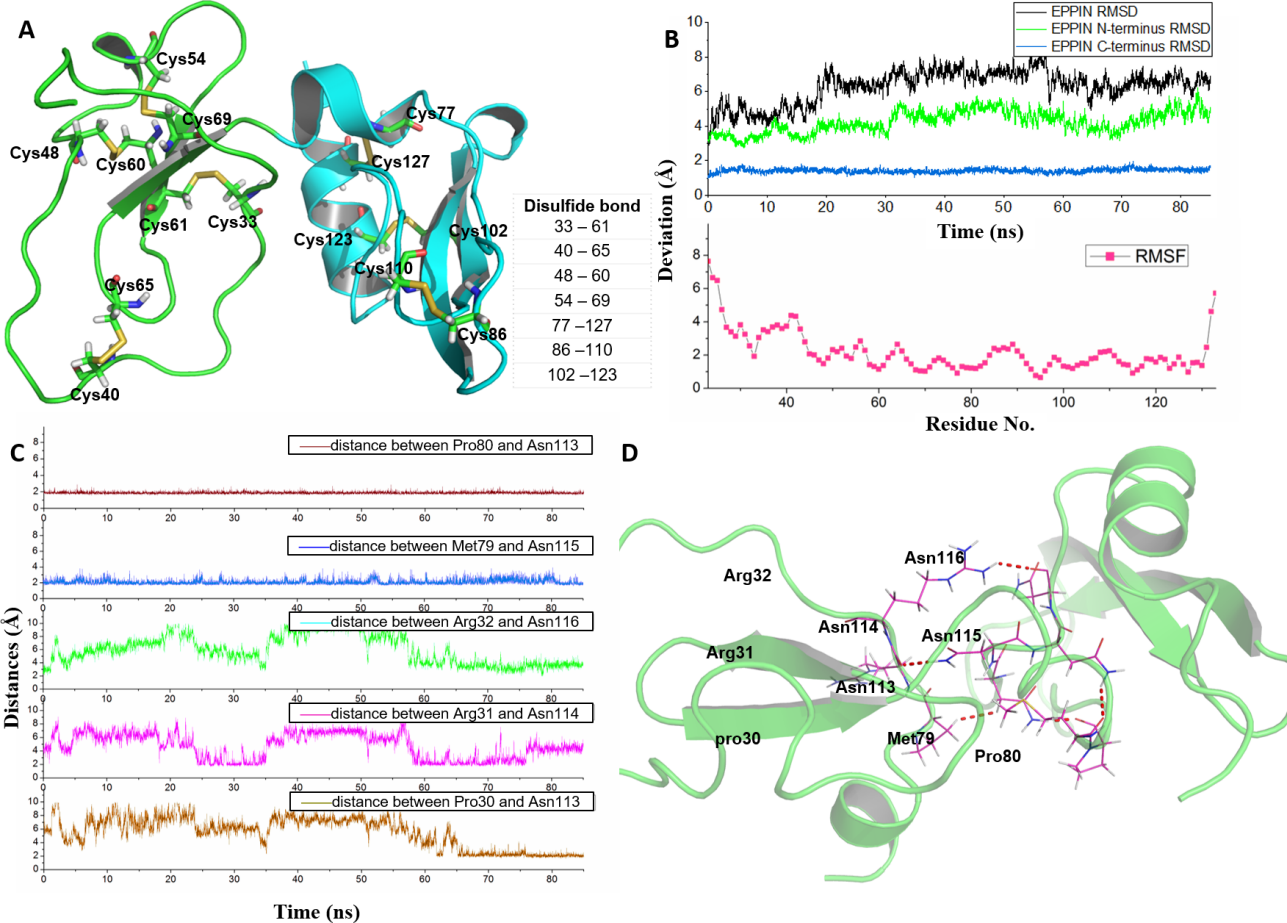
Homologous modeling structure of EPPIN after molecular dynamics stimulation. (A) The homology models of the N-terminus domain (23–77) and C-terminus domain (77–133) in EPPIN protein (disulfide bonds are highlighted as sticks); (B) RMSD (root mean square deviation, highlighted in black color) and RMSF (root mean square fluctuation, highlighted in red color) profile; (C) Time evolutions for the distances between the N-terminus (23–77) and C-terminus (77–133) of EPPIN, including Pro80 and Asn113, Met79 and Asn115, Arg32 and Asn116, Arg31 and Asn114, as well, Pro30 and Asn113. (D) The binding interaction between the N-terminus (22–77) and C-terminus (77–133) of EPPIN.

There were two β-sheet domains and four disulfide bonds in N-terminus, while C-terminus had similar structures to reported model (Silva et al., 2012) with two α-helix and two β-sheet domains (Fig. 1A). The quality of the EPPIN model was predicted by ProSA-web with value of −5 (Fig. S1) and Ramachandran plots (Fig. S2), indicated that this structure employed the good-quality in the range of the theoretical protein structure models.

In order to rationalize the 3D structure of EPPIN, 85 ns MD simulation with water and ion was performed. We found that the RMSD of EPPIN was equilibrated after 20 ns and was kept stable at 6 Å (in Fig. 1B). Because the disulfide bonds could stabilize the structure of EPPIN, the RMSF, a measurement of the average atomic mobility of their neighboring residues, had only a few changes with a deviation of 1–2 Å during the MD simulation (Fig. 1B). At the same time, the RMSF values of the residues in the N-terminus of EPPIN, especially, residues Gly23-Arg45, were much bigger than those in C-terminus, suggesting the C-terminus of EPPIN should have a smaller deviation. These results were consistent with the highly stable interactions between different residues in the C-terminus such as Pro80 and Asn113, as well Met79 and Asn115 (Figs. 1C and 1D) with distance of 2 Å.

Although the C-terminus is reported binding domain of SEMG1 (Silva et al., 2012), it is important to investigate the binding interactions between the C-terminus and N-terminus of EPPIN in order to understand the relationship between the structure and function among these domains. We found that some residues in the N-terminus, such as Arg31 and Arg32, were binding to Asn114 and Asn116 by hydrophobic interactions, respectively. In addition, there was a hydrogen bond between Pro30 and Asn113 with a distance of 2 Å. In brief, the disulfide bonds and the binding interactions between the C-terminus and N-terminus should stabilize the conformation of EPPIN.

### The molecular docking of SEMG1 peptide to EPPIN

**Figure 2:**
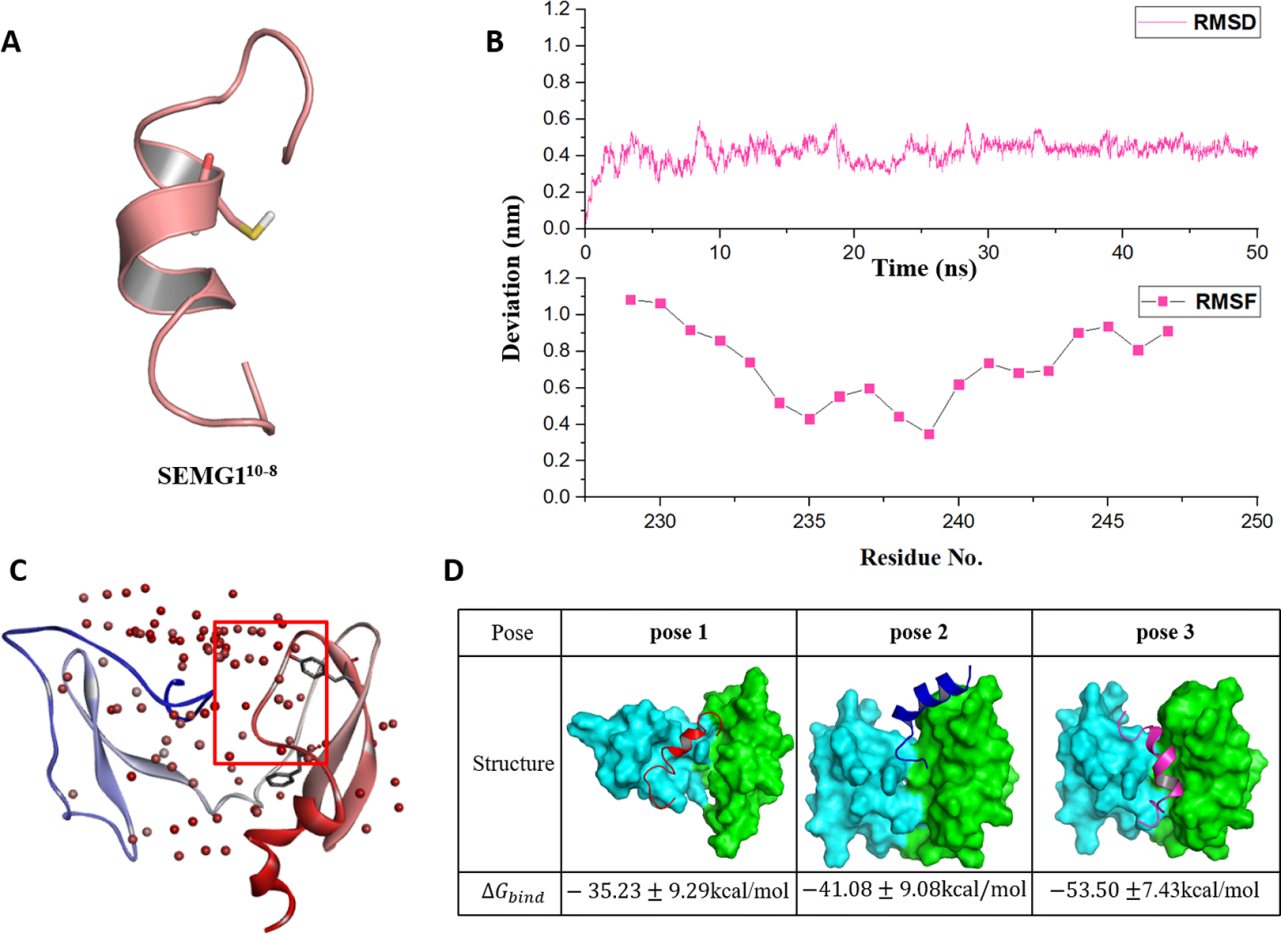
The molecular dynamics optimized strucrues of SEMG1^10-8^ peptide and the binding model of EPPIN and SEMG1^10-8^. (A) The 3D structure model of SEMG1^10-8^ peptide, Cys239 was shown as stick. (B) The RMSD, RMSF profile of SEMG1^10-8^ during the dynamic simulation. (C) The binding clusters of EPPIN. EPPIN was shown as cartoon and colored from blue (N-terminus domain) to red (C-terminus domain). Balls distributed over EPPIN indicates the binding cluster, the redder the balls, the more poses in the cluster. The red frame indicated the binding area, from which three binding poses were selected randomly. Tyr107 and Phe117 showed as sticks were utilized to filter the unwanted poses. (D) The three different poses for EPPIN and SEMG1^10-8^ complex and their binding free energies. The EPPIN protein in each pose was illustrated as molecular surface and colored by cyan (N-terminus domain) and green (C-terminus domain). SEMG1^10-8^ peptide was displayed as cartoon and shown as red, mazarine and megentas for each pose.

Seminal plasma protein semenogelin-1 has 462 residues, in which Cys239 is a critical amino acid for its binding to EPPIN. According to the truncation experimental data of the binding affinity with EPPIN (Silva, Hamil & O’Rand, 2013), the shortest SEMG1 peptide which could bind with EPPIN was SEMG1^10-8^ for the sequence of Glu229-Gln247. Thus, we chose this short SEMG1 peptide and constructed its structure by I-TASSER servers (Yang et al., 2015) (Fig. 2A). The most desirable model of SEMG1^10-8^ peptide got the *C*-score of −0.70. This score was typically in the range of −5–2, which is the quantitatively confidence of the models constructed by I-TASSER servers. A higher *C*-score signifies a model with a higher confidence and vice-versa (Yang et al., 2015). Moreover, this model displayed as an alpha helical structure, which was consistent with a previous report (O’Rand, Silva & Hamil, 2016).

Then, the 50 ns MD simulation for SEMG1^10-8^ with water and ion was performed. We found that the RMSD was equilibrated and stable after 10 ns for this peptide (Fig. 2B). Notably, Cys239, the irreplaceable residue for the binding of SEMG1 and EPPIN showed the lowest RMSF, indicated the binding site of SEMG1^10-8^ was more stable than the other part of this peptide (Fig. 2B).

The binding models of EPPIN with SEMG1^10-8^ peptide were generated by ZDOCK. The binding positions were initially filtered according to scores and the interactions with the key residues, such as Cys102, Tyr107 and Phe117 in EPPIN and Cys239 in SEMG1^10-8^. As shown in Fig. 2C, the most desirable binding center of SEMG1^10-8^ was located in the cleavage structure formed by the N- and C-terminus of EPPIN. Three binding poses for EPPIN-SEMG1^10-8^ were selected randomly in this most probable binding area for further MD studies (Fig. 2D). Further, the binding free energies calculated by MM/PBSA was adopted to filter the model for EPPIN-SEMG1^10-8^ complex. Besides considering the reported key residues of both SEMG1^10-8^ peptide (Cys239) and EPPIN (Tyr107 and Phe117) (Silva, Hamil & O’Rand, 2013; Silva et al., 2012), the binding model with the lowest free energy was selected as the most plausible model. As shown in Fig. 2D, the binding interaction of C-terminus of EPPIN and SEMG1^10-8^ was more reasonable because the corresponding **pose 2** and **pose 3** employed lower binding free energies (−41.08 kcal/mol and −53.50 kcal/mol, severally), while binding at N-terminus as **pose 1** resulted in −35.23 kcal/mol free energy (The binding free energy components was summarized in Table S1). These findings were consistent with previous reports (Silva et al., 2012), and further confirmed that SEMG1 possessed higher affinity to the C-terminus than N-terminus of EPPIN. Because **pose 3** had the lowest binding free energies, in which SEMG1^10-8^ occupied the groove near EPPIN C-terminus, we finally chose **pose 3** for further investigating the particular binding interaction of EPPIN and SEMG1^10-8^.

### Equilibration and stability of EPPIN-SEMG1^10-8^ complex

**Figure 3:**
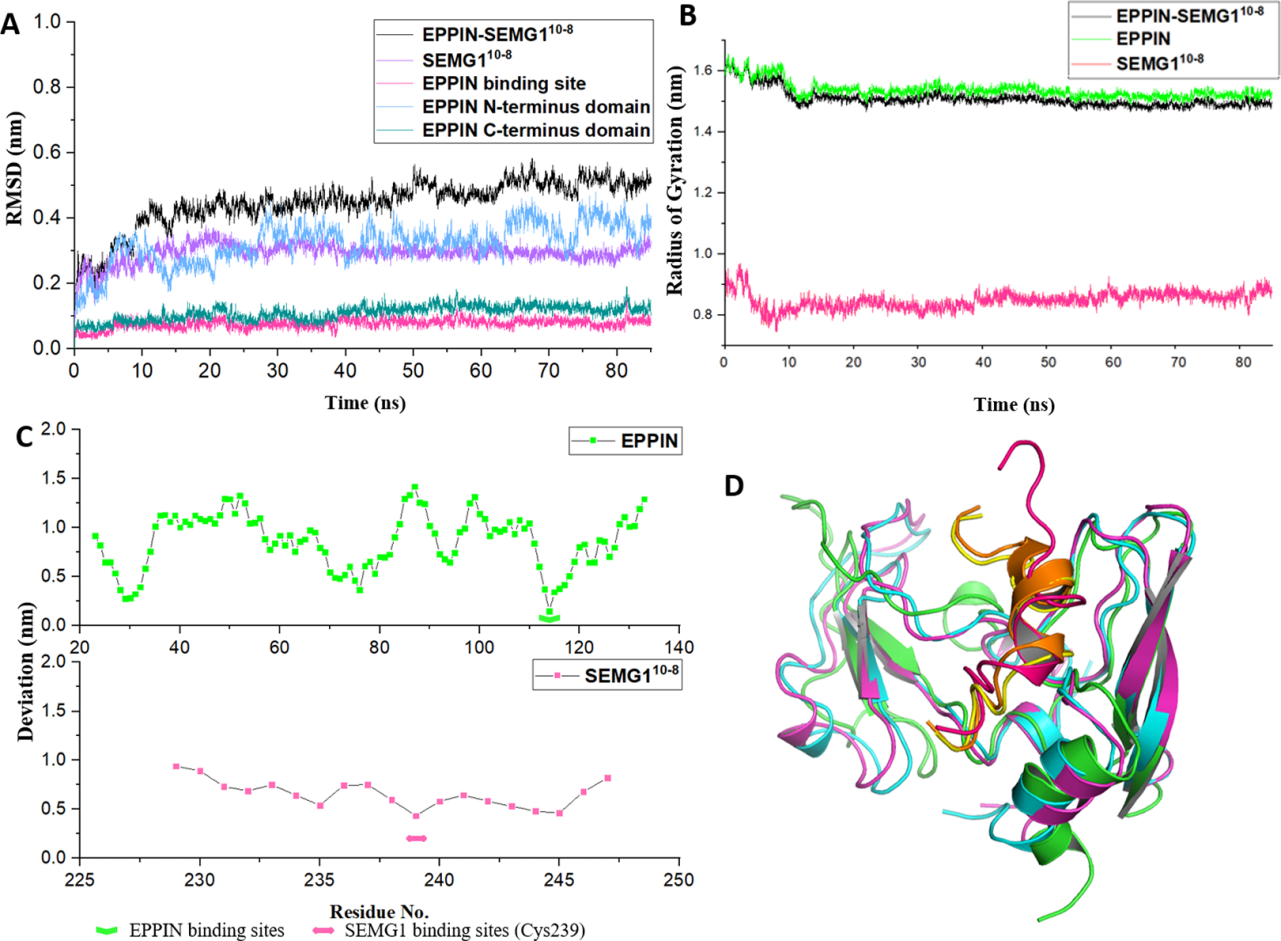
The molecular dynamic simulation process of EPPIN-SEMG1^10-8^ complex. (A) The RMSD of EPPIN-SEMG1^10-8^ complex, EPPIN N-terminus, EPPIN C-terminus, SEMG1^10-8^ and EPPIN binding site (Gln108-Asn122); (B) The evolution of the radius of gyration (*R*_g_) of the backbone (*C*α) atoms over the course of the simulation for EPPIN-SEMG1^10-8^ complex (black), EPPIN (green) and SEMG1^10-8^ (red); (C) The RMSF of EPPIN-SEMG1^10-8^ complex. The upper part (green dots line) indicated EPPIN component and the bottom part represented SEMG1^10-8^ (red dots line), respectively. The binding sites of EPPIN and SEMG1 in each complex were showed as fold lines and double-headed arrows severally; (D) Initial configuration superimposed on the last and the average configuration during 85 ns of simulation for EPPIN-SEMG1^10-8^ complex. Complexes are depicted as cartoons. For EPPIN, the initial, last and the average configuration were shown in green, magentas and cyan, respectively. For the SEMG1^10-8^ peptide, the initial, last and the average configuration were shown in red, yellow and orange, respectively.

As shown in Fig. 3A, the structure of the EPPIN-SEMG1^10-8^ complex gradually became stable during the first 20 ns simulation and the RMSD was about 5 Å after 20 ns. In the complex, the deviation of SEMG1^10-8^ component was about 3 Å after 20 ns. Moreover, the RMSD value of EPPIN N-terminus domain was about 3 Å and showed mild fluctuate. However, EPPIN C-terminus domain exhibited a much more stable and lower RMSD value (about 1 Å) which signified this domain remained significantly steady in the MD simulation process. Impressively, RMSD of the binding site of EPPIN (Gln108-Asn122) was quite low with the value of 0.5–1 Å, suggesting that the structure of EPPIN binding area was stable during the simulation.

Next, to study the conformational flexibility and compactness of the peptides and protein, their *R*_g_ values were calculated. The *R*_g_ of a set of atoms are the mass-weighted root mean square distance of those atoms from their center of mass (Davoudmanesh & Mosaabadi, 2018). The evolution of the *R*_g_ of the *C*α atoms in the process of the simulation was calculated for EPPIN, SEMG1^10-8^ as well as their complex. According to Fig. 3B, the *R*_g_ value showed a declining trend for EPPIN, SEMG1^10-8^ and their complex, indicating that the structural fluctuation of the protein complex became smaller along with the MD simulation time. Moreover, EPPIN presented slightly larger *R*_g_ values during the simulation than the EPPIN-SEMG1^10-8^ complex, which might result from the constricted main-chain movement and increased stability through the interaction of EPPIN and SEMG1^10-8^.

Further, the RMSFs of their *C*α atoms of the stabilized 60 ns were calculated to determine the conformational flexibility of EPPIN and SEMG1^10-8^ peptides. The residues of EPPIN binding site (Gln108-Asn122) exhibited a lower RMSF with the value of 2–3 Å (Fig. 3C), suggesting the binding site was rigid during the MD simulation. The similar trend could also be observed in the SEMG1^10-8^ peptide in the complex, residue Cys239, crucial for SEMG1 binding to EPPIN, represented the lowest RMSF (4 Å) (Fig. 3C). The fluctuations of the amino acids revealed that the binding sites were less flexible in both EPPIN and SEMG1^10-8^, proving that the interaction of EPPIN-SEMG1^10-8^ complex was stable during the MD simulation.

To elucidate the conformation changes during the simulation, the initial conformation of EPPIN-SEMG1^10-8^ complex was superimposed on the last conformation and the average conformation over the course of MD process (Fig. 3D). The last conformation of the SEMG1^10-8^ peptide was significantly different from its initial conformation, which exhibited a stable α-helix structure. Nevertheless, the last conformation was able to precisely align with the average conformation, especially for SEMG1^10-8^ and C-terminus of EPPIN, indicated the complex was particularly stable during the MD simulation.

### Interactions between EPIIN and SEMG1^10-8^

**Figure 4:**
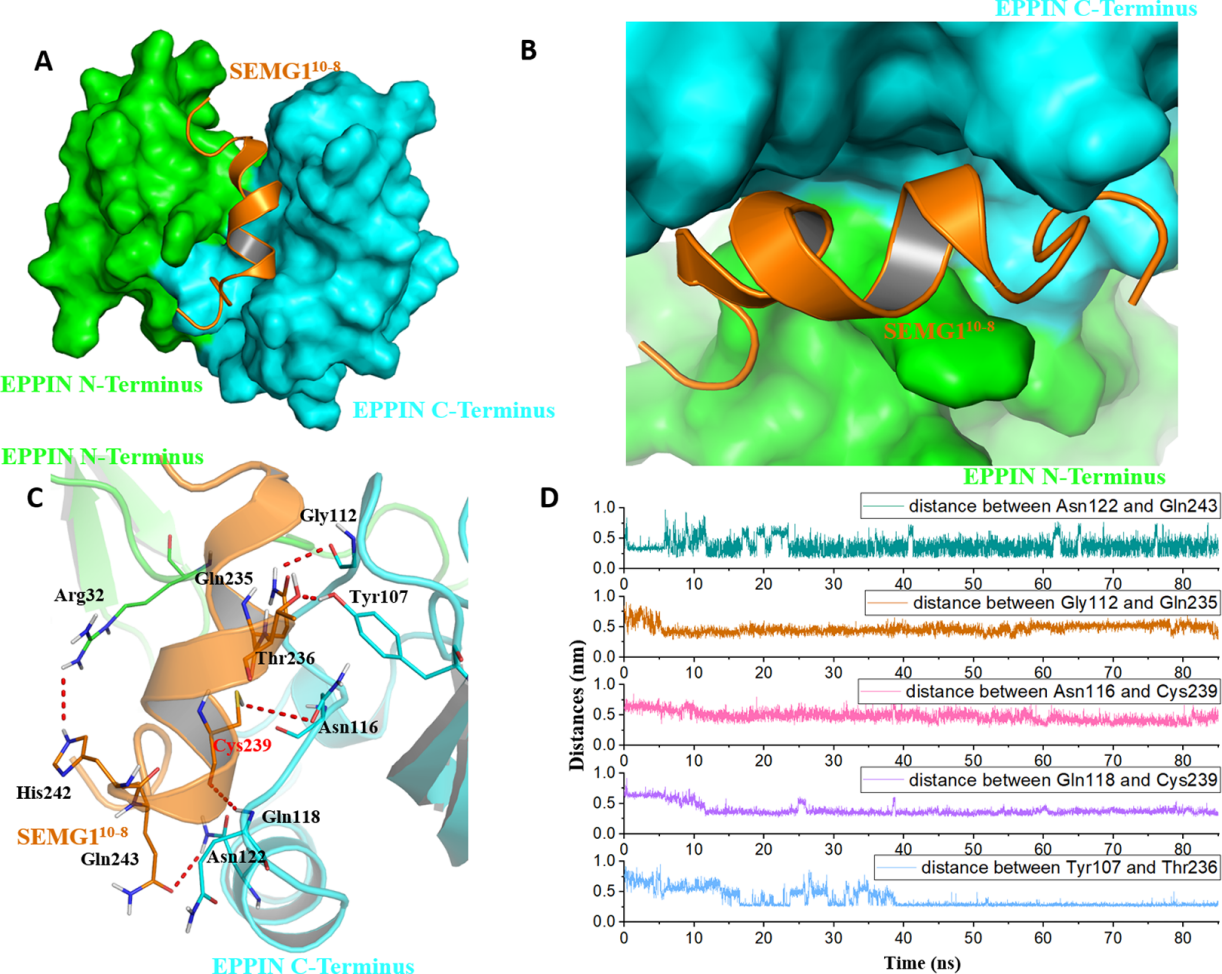
The binding interaction of EPPIN and SEMG1^10-8^. (A and B) The structure of EPPIN-SEMG1^10-8^ complex. EPPIN was depicted as protein surface in green (N-terminus domain) and cyan (C-terminus domai); (C) The binding interaction of EPPIN and SEMG1^10-8^. The important residues for the EPPIN-SEMG1 interactions were shown as lines and the hydrogen bonds between EPPIN and SEMG1 were illustrated by red dashed lines; (D) Time evolutions for the distances between EPPIN and SEMG1^10-8^, including Asn122 and Gln243, Gly112 and Gln235, Asn116 and Cys239, Gln118 and Cys239 as well as Tyr107 and Thr236.

Because the C-terminus domain was reported as the binding domain of SEGM1, the binding at this domain attracted most of the attention (Silva et al., 2012). As shown in Figs. 4A and 4B, SEMG1^10-8^ could bind with the C-terminus domain of EPPIN, which was consistent with the experimental results (O’Rand, Silva & Hamil, 2016). The MD simulation result showed that five important residues at the C-terminus domain had strong interactions with SEMG1^10-8^. It was found that these residues, including Tyr107, Gly112, Asn116, Gln118 and Asn122, stayed relatively close to the SEGM1^10-8^ peptide by hydrogen bonds with a short distance of 2–4 Å during the MD simulation (Figs. 4C and 4D). For the SEMG1 peptide, residues such as Gln235, Thr236, Cys239 and Gln243 were important for the formation of hydrogen bonds. Among these residues, Cys239 formed two hydrogen bonds with Asn116 and Gln118 of EPPIN, respectively, indicating Cys239 played a crucial role in the interaction between EPPIN and SEMG1, which confirmed the former report (Silva, Hamil & O’Rand, 2013). Unexpectedly, Arg32 in the N-terminus domain, which is an important amino acid for the interaction between the C- and N-terminus domains, could interact with His242 of SEGM1^10-8^.

In brief, we speculated that apart from the reported residues in C-terminus domain, such as Tyr107 and Gly112 (Silva et al., 2012), residues Asn116, Gln118 and Asn122 might play important roles for the binding of EPPIN. In addition, the interaction between the N-terminus domains of EPPIN with SEGM1 could not be ruled out due to these results.

### Important residues in the potential binding pocket of EPPIN

**Figure 5:**
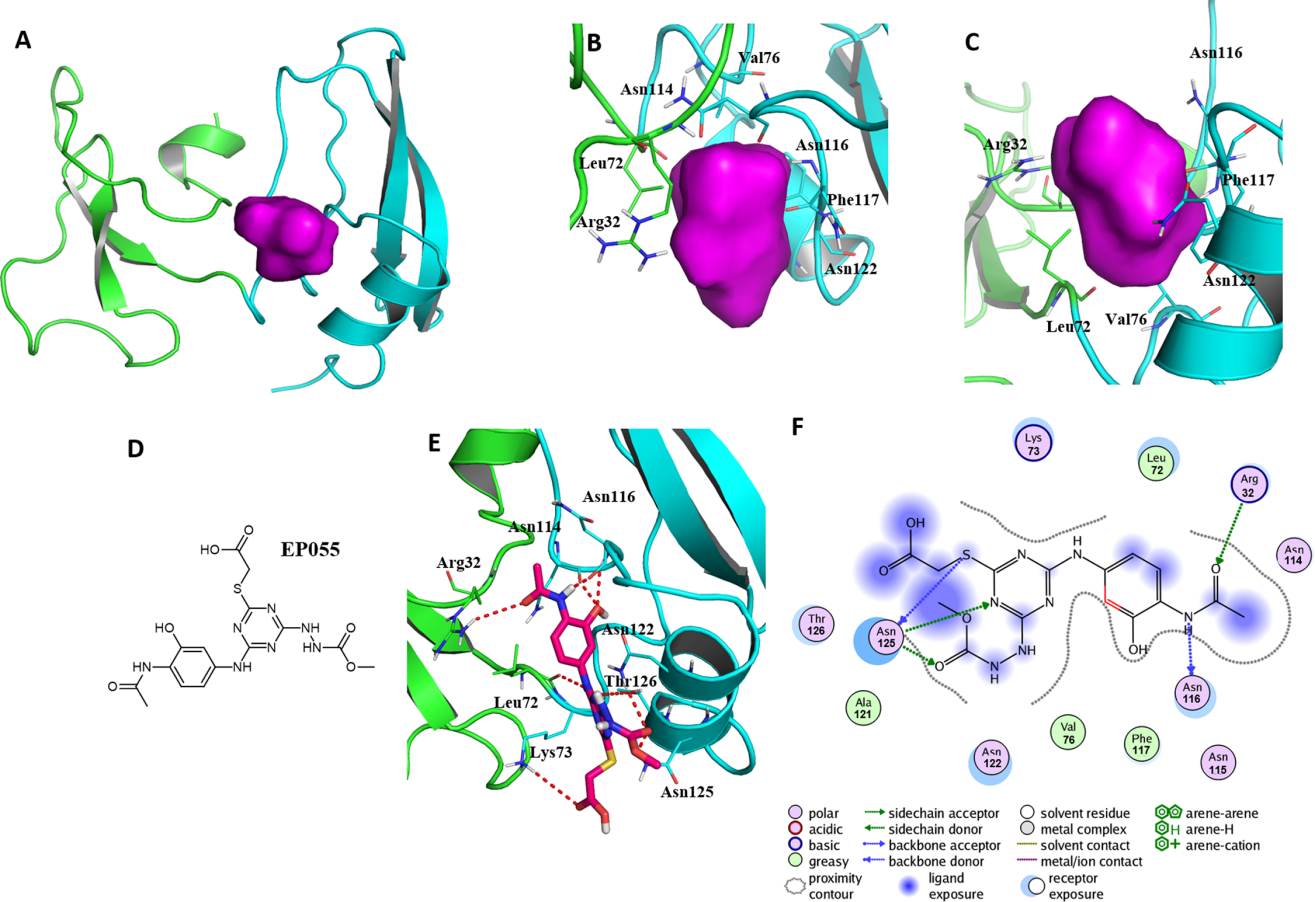
A reasonable potential binding pocket of EPPIN and the molecular docking of the reported ligand EP055 with EPPIN in this pocket. (A) The predicted binding pocket of EPPIN; (B) The dorsal, and (C) ventral view of the binding pocket. The important residues involved in the binding pocket for EPPIN are highlighted in green or blue bars; (D) The structure of **EP055**; (E and F) The 3D and 2D visualization and interaction within the EPPIN-EP055 complex. The important residues for the EPPIN and **EP055** interactions were shown as lines and the hydrogen bonds between EPPIN and **EP055** were illustrated by a red dashed line.

A reasonable potential binding pocket was generated by the SYBYL 2.0 Docking Suite module using the evaluated residues in the C-terminus of EPPIN for the binding of SEMG1, such as Tyr107, Gly112, Asn116, Gln118 and Asn122. As expected, this pocket of EPPIN was located mainly in the C-terminus. The residues involved in the binding pocket for EPPIN were highlighted in green or blue sticks (Figs. 5B and 5C), including Arg32, Leu72, Asn114, Asn116, Phe117 and Asn122. Some residues of the predicted pocket, for instance Phe117, were supported by the reported mutation data (Silva et al., 2012). Interestingly, the residues Arg32 and Leu72, which was located in N-terminus, were also found around the binding pocket. Other important residues, such as Asn116 and Asn122 in the potential binding pocket were consistent with the results of the molecule docking and MD simulations.

To further verify this binding pocket, molecular docking of the reported ligand **EP055** (O’Rand et al., 2018) (Fig. 5D) and EPPIN was conducted (docking score = 7.24, meaning a relatedly strong binding interaction). The structure of the EPPIN-EP055 complex showed that the **EP055** could form hydrogen bonds with Lys73, Asn114, Asn116, Asn122, Thr126 and Asn125 in the C-terminus of EPPIN. Impressively, the residues Arg32 and Leu72 in the N-terminus also exhibited two obvious hydrogen bonds with **EP055** (Figs. 5E and 5F). These results suggested that the **EP055** could bind with both terminuses of EPPIN by multiple binding interactions.

## Conclusions

The model of EPPIN was constructed by homological modeling based on the reported crystalline structures. The MD simulation results showed the structure of EPPIN was quite stable due to its disulfide bonds and the binding interaction between residues 113–116 in C-terminus domain and 30–32 in N-terminus domain. The model of SEMG1^10-8^ peptide, constructed by I-TASSER servers and rationalized by dynamics simulation, was docked with EPPIN. The binding free energy was calculated for different binding poses and the one with the lowest Δ*G*_bind_ was selected as EPPIN-SEMG1^10-8^ complex model. The binding interaction of SEMG1^10-8^ peptide and EPPIN was investigated by dynamics simulation. The results suggested that the C- and N-terminus domains of EPPIN were important for the binding of SEMG1. Additionally, we found the pocket formed by Arg32, Asn114, Asn116, Phe117 and Asn122 should be important for the design of new ligands of EPPIN. This detail binding interaction study might be helpful for the better understanding of the biological function of EPPIN and will encourage the discovery of non-hormonal contraceptive leads/drugs in the future.

## Supplemental Information

10.7717/peerj.7329/supp-1 Supplemental Information 1 The structure of EPPIN-SEMG1^10-8^ complex used for molecular dynamic simulation.

10.7717/peerj.7329/supp-2 Supplemental Information 2 The model quality of EPPIN and the molecular dynamics simulation box of EPPIN- SEMG1^10-8^ complex. Fig. S1: The protein sequence alignment between EPPIN C-terminus domain and different structure templates. Fig. S2: The model quality of EPPIN predicted by ProSA-web; Fig. S3: Ramachandran plots of EPPIN model; Fig. S4: The molecular dynamics simulation box of EPPIN- SEMG1^10-8^ complex; Table S1: Values of the binding free energy (kJ mol^−1^) and its components for three different binding poses of EPPIN-SEMG1^10-8^ complex calculated by MM/PBSA method; Table S2: Values of the binding free energy (kJ mol^−1^) and its components for three different binding poses of EPPIN-SEMG1^10-8^ complex calculated by MM/GBSA method.

## Additional Information and Declarations

### Data Availability

The following information was supplied regarding data availability: The raw data is available as a Supplemental File.
