# Supplementary material for: Binding interactions of epididymal protease inhibitor and semenogelin-1: a homology modeling, docking and molecular dynamics simulation study
Source: PeerJ. 2019 Aug 5;7:e7329. doi: 10.7717/peerj.7329 (PMC6686837; doi:10.7717/peerj.7329)
Supplement: Supplemental Information 2 — Fig. S1: The protein sequence alignment between EPPIN C-terminus domain and different structure templates. Fig. S2: The model quality of EPPIN predicted by ProSA-web; Fig. S3: Ramachandran plots of EPPIN model; Fig. S4: The molecular dynamics simulation box of EPPIN- SEMG110-8 complex; Table S1: Values of the binding free energy (kJ mol−1) and its components for three different binding poses of EPPIN-SEMG110-8 complex calculated by MM/PBSA method; Table S2: Values of the binding free energy (kJ mol−1) and its components for three different binding poses of EPPIN-SEMG110-8 complex calculated by MM/GBSA method. [file peerj-07-7329-s002.docx]

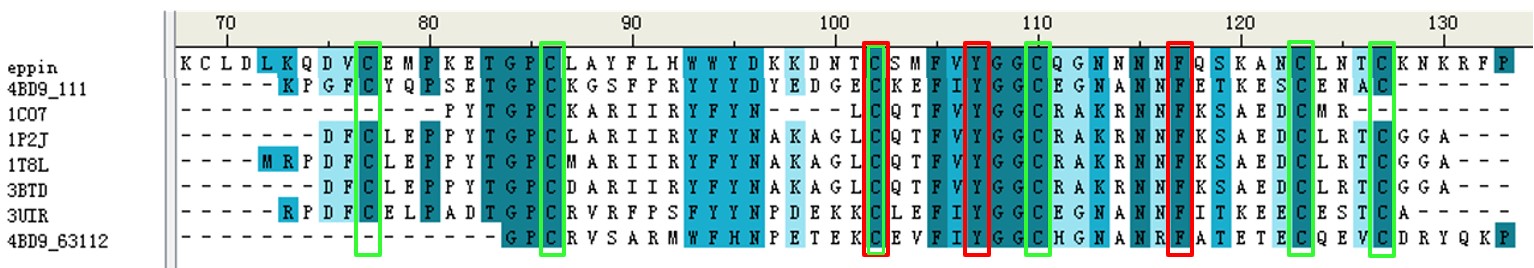


**Figure S1.** The protein sequence alignment between EPPIN C-terminus domain and different structure templates. The red frames indicated the important residues of EPPIIN C-terminus domain for its binding to SEMG1 protein and the green frame represented the conserved cysteines which could from the disulfide bonds. The sequence identity and sequence similarity between EPPIN C-terminus domain (first line) and carboxypeptidase inhibitor SmCI (PDB ID: 4BD9, chain B) (second line) was 29.4% and 45.1% respectively.


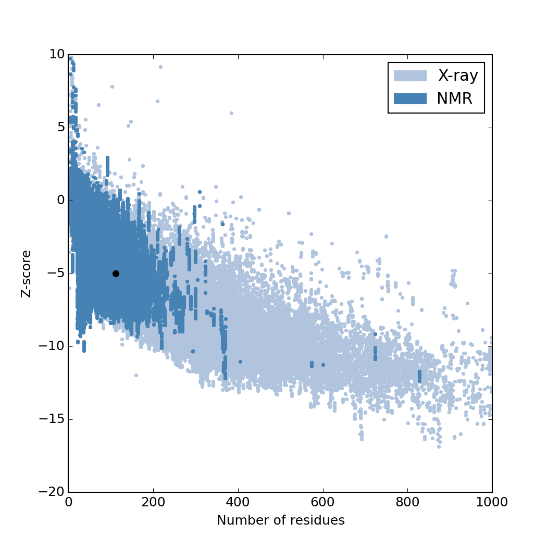

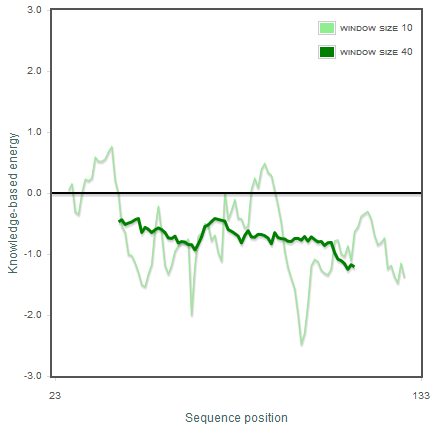


(A) (B)

**Figure S2.** (A) Overall model quality and (B) local model quality of EPPIN model predicted by ProSA-web. In the figure of overall model quality, black dot represented our EPPIN model, showing the model had the quality as the NMR structures.


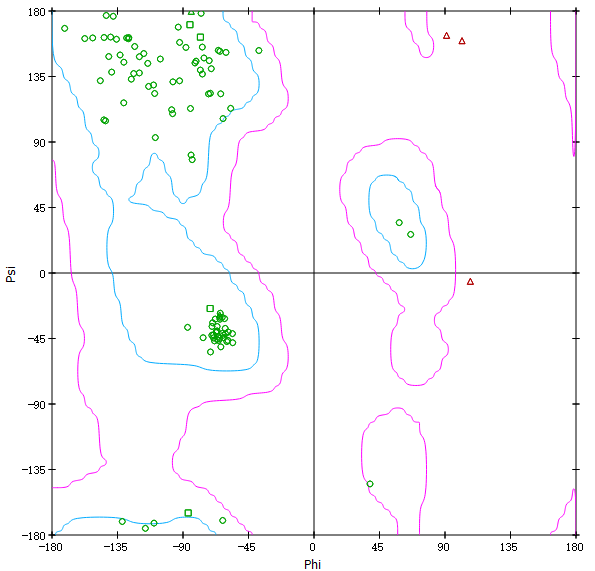


**Figure S3.** Ramachandran plots of EPPIN model. Residues marked by green showed that these residues had the excellent or acceptable angles; however, residues marked by red meant that angles were not acceptable. For our model, only three residues' angle were not good, but these residues were not involved in the binding pocket. Moreover, even the crystal structures also had some residues with bad angles.

**
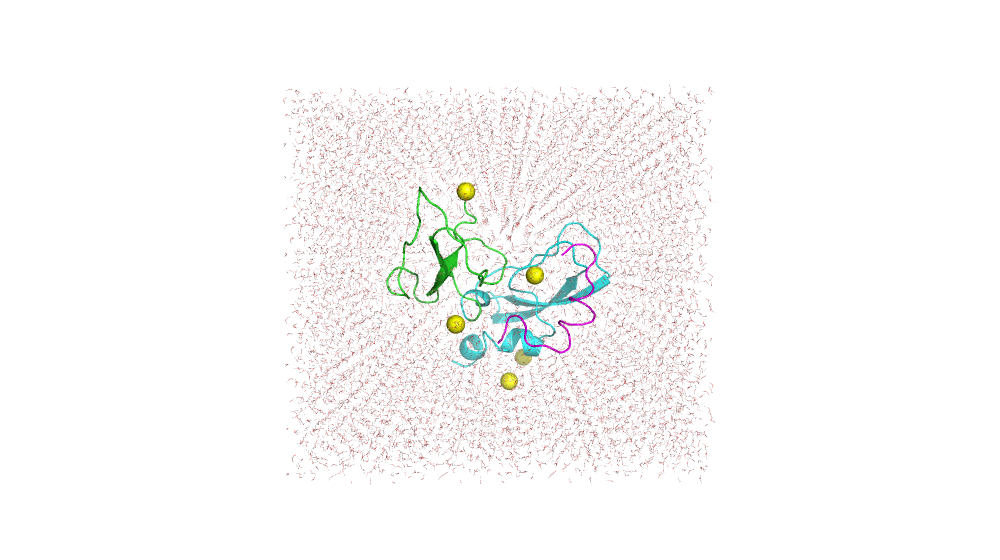
**

**Figure S4.** The molecular dynamics simulation box of EPPIN- SEMG1^10-8^ complex with chloride ion (yellow balls) and water. EPPIN was colored with green and cyan from N-terminal to C-terminal, SEMG1^10-8^ was colored with magenta.

**Table S1.** Values of the binding free energy (kJ mol^−1^) and its components for three different binding poses of EPPIN-SEMG1^10-8^ complex calculated by MM/PBSA method

| Pose | $\Delta E_{int}$ | $\Delta E_{vdw}$ | $\Delta E_{ele}$ | $\Delta G_{polar}$ | $\Delta G_{SA}$ | $\Delta G_{bind}$ |
| --- | --- | --- | --- | --- | --- | --- |
| **Pose1** | 0.00(0.00) | -43.84(4.93) | -200.69(37.50) | 215.06(32.87) | -5.85(0.50) | -35.32(9.29) |
| **Pose2** | 0.00(0.00) | -87.02(6.94) | -195.80(39.74) | 250.89(39.23) | -9.15(0.46) | -41.08(9.08) |
| **Pose3** | 0.00(0.00) | -54.35(7.43) | -275.64(40.41) | 283.30(40.46) | -6.81(0.59) | -53.50(7.43) |

Standard deviations are reported in parentheses. ΔE_int_ is the internal energy related to bond, angle, and dihedral parameters, ΔE_ele_ is the electrostatic energy, ΔE_vdw_ is the van derWaals energy, ΔG_polar_ is the electrostatic solvation energy, and ΔG_SA_ is the non-electrostatic solvation energy. ΔGbind was calculated via the following equation: ΔG_bind_ =ΔE_int_ +ΔE_ele_ +ΔE_vdw_ +ΔG_polar(PB)_ +ΔG_SA_

**Table S2.** Values of the binding free energy (kJ mol^−1^) and its components for three different binding poses of EPPIN-SEMG1^10-8^ complex calculated by MM/GBSA method

| Pose | $\Delta E_{vdw}$ | $\Delta E_{ele}$ | $\Delta G_{polar}$ | $\Delta G_{non-polar}$ | $\Delta G_{bind}$ |
| --- | --- | --- | --- | --- | --- |
| **Pose1** | -43.84(4.93) | -200.69(37.50) | 217.75(33.83) | -7.16(0.79) | -33.95(8.65) |
| **Pose2** | -87.02(6.94) | -195.80(39.74) | 243.72(38.14) | -11.87(0.85) | -50.98(8.21) |
| **Pose3** | -54.35(7.43) | -275.64(40.41) | 294.50(38.78) | -8.70(0.87) | -44.19(6.35) |

Standard deviations are reported in parentheses. ΔE_ele_ is the electrostatic energy, ΔE_vdw_ is the van derWaals energy, ΔG_polar_ is the electrostatic solvation energy, and ΔG_non-polar_ is the non-electrostatic solvation energy. ΔGbind was calculated via the following equation: ΔG_bind_ = ΔE_ele_ +ΔE_vdw_ +ΔG_polar(GB)_ +ΔG_non-polar_
